# Assembling the *Setaria italica* L. Beauv. genome into nine chromosomes and insights into regions affecting growth and drought tolerance

**DOI:** 10.1038/srep35076

**Published:** 2016-10-13

**Authors:** Kevin J. Tsai, Mei-Yeh Jade Lu, Kai-Jung Yang, Mengyun Li, Yuchuan Teng, Shihmay Chen, Maurice S. B. Ku, Wen-Hsiung Li

**Affiliations:** 1 Bioinformatics Program, Taiwan International Graduate Program, Institute of Information Science, Academia Sinica, Taipei, 11574 Taiwan; 2 Institute of Biomedical Informatics, National Yang-Ming University, Taipei, 11221 Taiwan; 3 Biodiversity Research Center, Academia Sinica, Taipei, 11574 Taiwan; 4 Department of Bioagricultural Science, National Chiayi University, Chiayi, 60004 Taiwan; 5 School of Biological Sciences, Washington State University, Pullman, WA 99164, USA; 6 Department of Ecology and Evolution, University of Chicago, Chicago, IL 60637 USA

## Abstract

The diploid C_4_ plant foxtail millet (*Setaria italica* L. Beauv.) is an important crop in many parts of Africa and Asia for the vast consumption of its grain and ability to grow in harsh environments, but remains understudied in terms of complete genomic architecture. To date, there have been only two genome assembly and annotation efforts with neither assembly reaching over 86% of the estimated genome size. We have combined *de novo* assembly with custom reference-guided improvements on a popular cultivar of foxtail millet and have achieved a genome assembly of 477 Mbp in length, which represents over 97% of the estimated 490 Mbp. The assembly anchors over 98% of the predicted genes to the nine assembled nuclear chromosomes and contains more functional annotation gene models than previous assemblies. Our annotation has identified a large number of unique gene ontology terms related to metabolic activities, a region of chromosome 9 with several growth factor proteins, and regions syntenic with pearl millet or maize genomic regions that have been previously shown to affect growth. The new assembly and annotation for this important species can be used for detailed investigation and future innovations in growth for millet and other grains.

*Setaria italica*, commonly known as foxtail millet, is the second-most widely planted species of millets in the world and an important grain crop model^1^,^2^,^3^. It is widely grown as a grain or forage crop in temperate and subtropical Asia and parts of southern Europe, North America, South America, Australia and North Africa. Cultivation of the species dates back to 5000–6000 BC in China^4^, where the species can grow under harsh, arid environments. However, despite its biological and agricultural importance, the species remains understudied in terms of genomic architecture, partly because of its lack of popularity due to low nutritional value^5^, lack of iodine and indigenous selectivity to specific parts of Africa and Asia, and partly because of the lack of a complete assembly and annotation.

From a genomics perspective, there have been several mappings of genetic markers for foxtail millet^6^,^7^ and even physiological QTL mappings for its close relative pearl millet^8^. However, studies on the genetic architecture of drought tolerance and biomass are limited and currently confined to cross-species studies^9^,^10^. To date, there have only been two published genome assemblies and annotations of foxtail millet, neither of which had reached over 86% of the estimated genome size^11^,^12^. The two publications referenced different estimated genome sizes for foxtail millet (490 Mbp and 510 Mbp), but we have chosen to use 490 Mbp throughout this work because it is a more recent reference^12^.

In Taiwan, foxtail millet is popular in the eastern Taitung County, where it is used to produce a specialty wine, and the current commercial cultivar is called TT8. We have recently isolated a foxtail millet mutant from a sodium-azide mutagenized mutant pool that allows the panicle of the millet to grow longer than the wild type and in a greater grain number (Figure S1). On average, the mass of the crop yield is up to two times heavier than that of the wild type.

The goal of this study is two-fold. The first is to assemble and annotate a more complete version of the foxtail millet genome as past assemblies have only completed 83–86% of the genome. A good assembly can facilitate a more complete collection of candidates of the mutation(s) that produces the larger panicles and greater grain yield mentioned above. The second is to characterize and understand more about the genetics of the growth and drought tolerance of TT8, either in previously unassembled regions or from sequence variations and mutations. Recent studies on foxtail millet population genetics^13^ and comparative studies^14^ have already benefited from previous assembly efforts, where the assemblies are used as references for mapping and variant determination. In future studies, the identification of the mutation(s) that produce larger panicles and discovery of potential to transfer those desirable traits to cereals with better nutritional value would make a significant contribution to our rapidly growing population.

## Results

### Assembly

To construct a good assembly of the foxtail millet genome we obtained various deep sequencing data for high-resolution assembly and scaffolding, including Illumina paired-end reads (PE), mate-pair reads (MP), and synthetic long reads (SLR), and Roche 454 long distance paired-end reads (454) (Tables S1–S4). After processing the raw reads (see Methods) we obtained a total coverage depth of 377x genome length based on the assembled 477 Mbp genome length, including 95x coverage from PE reads used in contig assembly with a 85% FLASH^15^ merging overlap and the remaining 282x coverage used for assembly improvement (scaffolding, gap filling, etc.).

**Figure 1:**
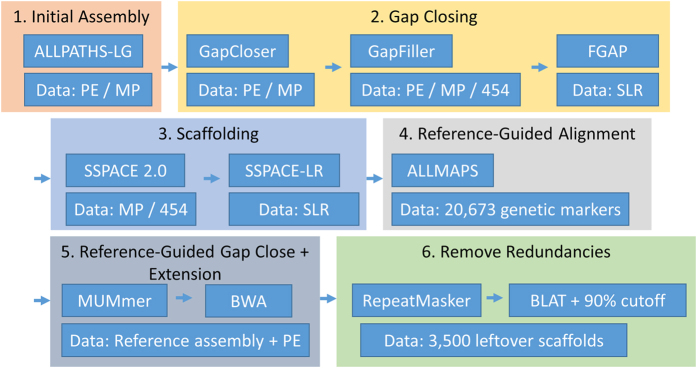
Workflow for assembly of the foxtail millet TT8 genome. Each of the post-assembly improvement steps involved the use of multiple bioinformatics tools. Listed under each tool name is the data used with the tool. The data for a tool was selected according to the best post-improvement results, availability at the current stage, and the best fit with the applied tool.

With the PE and MP reads we calculated an estimated genome size of 433 Mbp using ALLPATHS-LG^16^ and reached an initial assembly of 410 Mbp. However, it has been previously estimated experimentally that the total foxtail millet genome length is 490 Mbp^12^. In order to surpass our initial genome size limit we created a post-assembly improvement workflow that brought the TT8 assembly to 477 Mbp (Fig. 1), with 98% of the PE reads mappable back to the final assembly.

Although we conducted several post-assembly improvement steps, most steps yielded minor improvements in terms of assembly statistics. The two major improvement steps were genetic marker rearrangements via ALLMAPS^17^ and a custom reference-guided extension and gap closing approach we applied using leftover scaffolds and PE reads. For the reference assembly, we used the assembly of the Joint Genome Institute (JGI) version JGI 8x v2 Sitalica_164 (ftp://ftp.jgi-psf.org/pub/JGI_data/phytozome/v8.0/Sitalica/). JGI’s assembly is of high quality, nearly 83% complete assembly with the nine chromosome scaffolds established and a smaller number of remaining scaffolds and gaps^11^.

**Figure 2:**
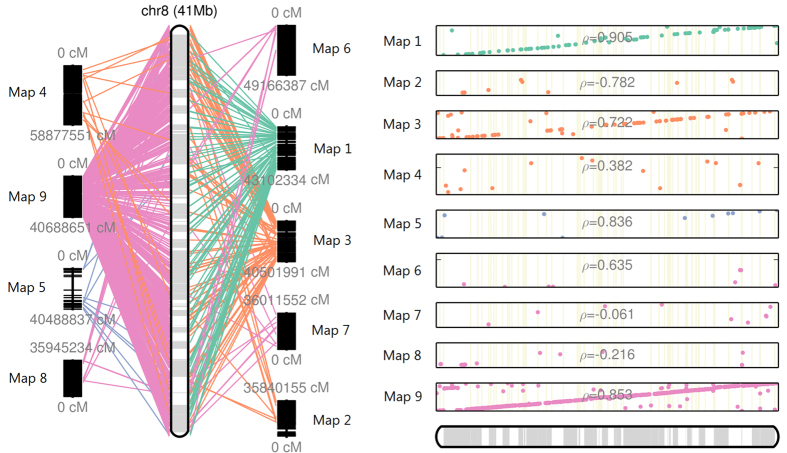
Creation of chromosome 8 via scaffolds, genetic markers, and ALLMAPS. *Left*, the 9 black sequences (vertical bars) adjacent to chromosome 8 represent the genetic marker maps that anchored our assembly scaffolds. The lines in pink, orange, and green represent genetic markers from different marker maps. Each map has a different number of markers at various densities as shown in the black lines stacked in vertical bars. Most of the information used to construct chromosome 8 came from a single input map with a highly linear marker alignment as illustrated in pink. The other two input maps with a strong influence on the construction of chromosome 8 are shown in green and orange. Interestingly, some of the input maps align in reverse order to the genome position (e.g., the bottom left map and the two bottom right maps) but lacking a strong influence on the construction of the chromosome. *Right*, the tracks show the alignment between marker and scaffold placement, in most cases displaying a linear marker order consistency.

For the ALLMAPS step, we collected 749 markers from the Beijing Genomics Institute (BGI)^12^ and 19,924 markers from the National Institute of Plant Genome Research of New Delhi, India^18^. We then used BLAST^19^ to map the markers onto our existing scaffolds and arranged the scaffolds with ALLMAPS according to the marker’s original location. This led to the arrangement and contiguation of several scaffolds while increasing the number of N’s by ~65 kbp. The TT8 assembly went from an initial assembly with N50 = 1,774,338 bp and 4,194 scaffolds (maximum scaffold length of 10,280,153 bp) to the improved one with N50 = 43,352,192 bp and 3,558 scaffolds (maximum scaffold length of 60,135,426 bp) (Table S5). In total, 648 scaffolds consisting of 94.9% of the assembly were anchored onto 9 chromosomes (Fig. 2). Of those, 172 scaffolds were organized in a co-linear orientation, 256 scaffolds had an unknown orientation, and 220 scaffolds were oriented in an anti-colinear fashion. Of the 256 scaffolds with unknown orientation, 238 scaffolds had only 1 genetic marker mapped causing an undeterminable orientation.

To improve the TT8 assembly, we originally used a published reference-guided scaffolding program, called Scaffold_Builder^20^, but we found that our non-chromosome leftover scaffolds and PE reads would map to repeat regions of the genome instead of making extensions, scaffolds, or filling gaps. Instead, we built a custom reference-guided improvement program that extended large (>400 kbp) gaps. The basis for the custom pipeline is essentially a multiple sequence alignment using MUMmer^21^ and BWA^22^ between the TT8 assembly and the JGI 8x v2 Sitalica_164 assembly as a reference, and using leftover scaffolds and PE reads from the previous steps. With the leftovers mapped to the JGI 8x v2 Sitalica_164 reference, and the reference mapped to places where we had gaps, we were able to align many remaining scaffolds and PE reads to the TT8 assembly.

**Figure 3:**
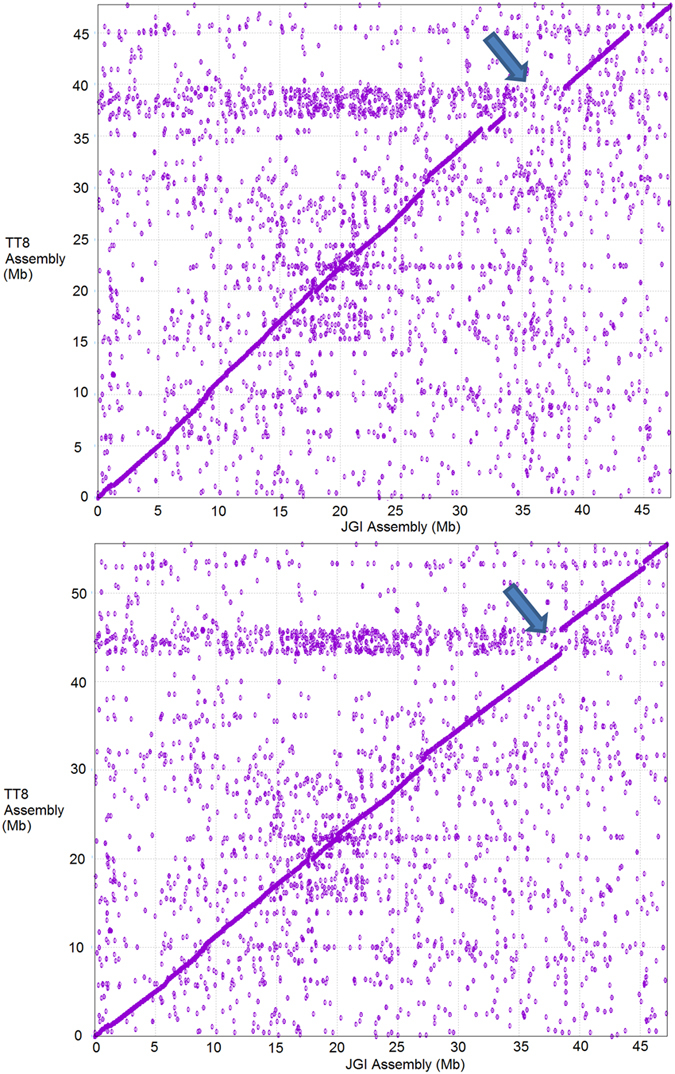
MUMmer plot comparison of before (top) and after (bottom) reference-guided extension for chromosome 5. The x-axis represents our assembled chromosome 5 and the y-axis represents JGI 8x v2 Sitalica_164 chromosome 5. The top panel showed that there were 5 regions over 400 kb in length that were present in JGI 8x v2 Sitalica_164 but not in TT8, with the most noticeable gap at the 33 Mbp to 38 Mbp x-axis range (indicated by a blue arrow). The bottom panel shows the final assembly that the horizontal gap has now been closed by an extension at 33 Mbp assembled by the leftover scaffolds and PE reads. The vertical gap is unchanged as it represents a portion of chromosome 5 absent in JGI 8x v2 Sitalica_164.

For assembly extensions, we identified 28 regions in the JGI 8x v2 Sitalica_164 assembly that were missing in TT8 with a minimum length of 400 kbp; Chr 5 is shown as an example in Fig. 3. We then made an insertion of leftover scaffolds and PE reads using the same multiple alignment approach. We were able to reduce the number of N’s from 20,961,137 bp to 7,847,864 by gap closure, and we finally increased the total length of the assembly from 415,411,431 bp to 479,782,239 bp by reference-guided extension (Table S5).

Compared to the JGI 8x v2 Sitalica_164 assembly and the BGI assembly Millet_scaffoldVersion2.3 (ftp://ftp.genomics.org.cn/pub/Foxtail_millet/genome_assembly/Millet_scaffoldVersion2.3.fa.gz), our TT8 assembly is substantially more complete in terms of total length (relative to the estimated genome size of 490 Mbp) with a much lower number of N bases, leading to longer lengths of N50, N75 and N95 (Table 1). In view of the difference in total length between the TT8 assembly and the JGI 8x v2 Sitalica_164 assembly (total 477 vs. 405 Mbp), the difference between L50 = 5 (this study) and L50 = 4 (JGI 8x v2 Sitalica_164) is insignificant. An L95 = 9 means that over 95% of our genome draft is mapped to the nine chromosomes and each of our individual chromosomes is longer in length than the JGI 8x v2 Sitalica_164 counterpart. We also ran CEGMA to verify genome completeness using known ultra-conserved genes^23^, and found the results among all three assemblies to be approximately the same. It should be noted that there is one area where the JGI 8x v2 Sitalica_164 assembly is better, which is in the total number of scaffolds and gaps, thanks to the native large contig nature of BAC sequencing compared to our Illumina PE reads (Table 1).

Our initial assembly with ALLPATHS-LG alone did not generate an assembly that was more complete than those of JGI’s 8x v2 Sitalica_164 and BGI’s Millet_scaffoldVersion2.3. Several repeat regions posted an obstacle for the assembler that was also noted by others^11^,^12^,^24^. The obstacle could be one reason why assembly attempts until now had only reconstructed ~85% of the genome and explains our 4% increase in maskable repeat content over Millet_scaffoldVersion2.3 (Table S6). Nevertheless, there is definitely novel information in the remaining 15% that is not repeats as shown by our annotation results below.

### Annotation

#### Overall annotation statistics

For our annotation analysis we primarily focused on transcripts, proteins and functional annotation in comparison with JGI 8x v2 Sitalica_164 and BGI Millet_scaffoldVersion2.3 (Table 2). Our initial gene prediction from running MAKER2^25^ with SNAP^26^ and GeneMark^27^ predicted 44,292 gene models in the TT8 assembly. Among the predicted gene model hits, 91.5% were annotated to plant genes in millet, maize, switchgrass and rice. After additional efforts of contamination removal with adaptor trimming and screening (see Methods), the remaining 8.5% gene models were mapped to fungi, animals and/or bacteria. These non-plant gene models were located in the 9 chromosome scaffolds and did not replace any of the non-chromosome scaffolds (i.e., orphan fragments not anchored to the TT8 assembly’s chromosomal scaffolds). The 44,292 predicted gene models cover over 10% length of the genome draft (49 Mbp of 477 Mbp). We used BLAST with NCBI’s non-redundant (nr) database and Pfam^28^ to collect hits and significance values among assemblies. Our number of predicted genes and functional annotation unique terms are all greater than those in JGI 8x v2 Sitalica_164 and BGI Millet_scaffoldVersion2.3 (Table 2).

Although an earlier study^11^ reported only 24,000~29,000 annotated genes in JGI’s dataset, their annotated sequence files contained 40,599 predicted transcripts and proteins. This is due to their conservative qualification of a fully annotated gene model based on a high confidence statistic and filtering of genes that do not align to a known grass gene^11^. Searching by BLAT alignment^29^ with our predicted transcripts as queries, we found our transcripts aligned and covered 33,259 of JGI 8x v2 Sitalica_164′s 40,599 predicted transcripts (82%). Among those, 11,156 of TT8 transcripts shared over 99% similarity to their homologs.

#### Annotation of the miscellaneous non-chromosome scaffolds

The remaining 2,679 un-anchored scaffolds contained 1,366 predicted genes, only 640 of which had putative transcript length over 500 bp. They accounted for slightly over 1% of the total number of predicted genes and were lower in transcript to DNA proportion than the rest of the genome. In other words, nearly 99% of the available annotation information is harbored in our nine assembled chromosomes.

#### Gain and loss of functional annotation across genome and newly assembled regions

In terms of GO term annotation (Table 2), the three assemblies each contain their own unique terms, with nearly half of the unique terms being exclusive to the TT8 assembly (>800 GO terms unique to this study compared to 230 unique to other assemblies) (Figure S2). Of the TT8 assembly’s novel GO terms (not found in BGI Millet_scaffoldVersion2.3 or JGI 8x v2 Sitalica_164), there is a proportionally larger number in the cellular component and biological process categories. Among those, 20% of the biological process terms are related to metabolism of varying compounds such as single-carbon compounds, mRNA, carbohydrates, fats, and sugars. The pathways involved include glycolysis, gluconeogenesis and the TCA cycle. This pattern is seen both across the genome and in the functional annotation of newly assembled regions. In particular, protein geranylation is enriched in both molecular and biological process GO categories (Figure S3). GGTases are involved in addition of geranyl groups to aromatic compounds in higher plants. These compounds, originating from several natural product classes, often hold antimicrobial, antioxidant, anti-inflammatory, antiviral, and anticancer activities^30^.

Significantly, the TT8 assembly contains 6 novel terms specifically related to growth and growth factors that are absent from JGI 8x v2 Sitalica_164 and Millet_scaffoldVersion2.3, among which four terms are in biological processes (GO:0040007, GO:0006020, GO:0031929, GO:0016049) that are crucial for a more complete understanding of millet growth. Specifically, these terms are related to size of cell and organ, inositol biosynthesis, response to nutrition availability, and cell growth rate. In particular, response to nutrition availability and inositol biosynthesis are important for its ability to grow under harsh, arid environments. Enhanced myo-inositol biosynthesis in a number of plants is correlated with increased resistance to biotic (e.g., insect) and abiotic (e.g., drought, salinity) stress conditions^31^, consistent with the notion of foxtail millet being one of the most drought tolerant C_4_ cereals^32^. Of the terms missing in the TT8 assembly that are unique to JGI 8x v2 Sitalica_164 and Millet_scaffoldVersion2.3, 20 unique terms (9% of the total 230) are annotated as obsolete as of the date of the database query (10/01/2015). Although the updated versions of these obsolete terms may account for 20 unique TT8 GO terms, they pale in comparison to the >800 total found in TT8. Overall the TT8 assembly is 73 Mbp longer than JGI 8x v2 Sitalica_164. There are 2–5 newly assembled contiguous regions per chromosome in the TT8 assembly that were identified as large gaps (over 400 kbp) in the JGI 8x v2 Sitalica_164 assembly during reference-guided extension by MUMmer alignment. In those regions, there were 227 newly predicted genes and 95 unique GO terms. Although there is an even distribution of 2–5 newly assembled regions on each scaffold, most newly predicted genes from regions absent in the JGI 8x v2 Sitalica_164 assembly were found in chromosomes 3, 5 and 6 (Figure S4).

#### Candidate genes based on known ontology families

Previous efforts on Millet_scaffoldVersion2.3 described a gene ontology family, ORTHOMCL4, containing GO terms related to response under stress^12^. In the Millet_scaffoldVersion2.3 assembly, the 4 GO terms (GO:0006950; response to stress; Biological Process; GO:0009415; response to water; Biological Process; GO:0006508; proteolysis; Biological Process; GO:0008234; cysteine-type peptidase activity; Molecular Function) related to 586 candidate genes. These GO terms are part of the shared GO terms with the TT8 assembly, and TT8 has 842 predicted gene models related to those terms. It will be difficult to compare the differences between gene models in Millet_scaffoldVersion2.3 and TT8 without the list of 586 Millet_scaffoldVersion2.3 genes, however, our 842 predicted gene models can be noted as candidates for further studies.

#### QTL and marker comparison with pearl millet

**Figure 4:**
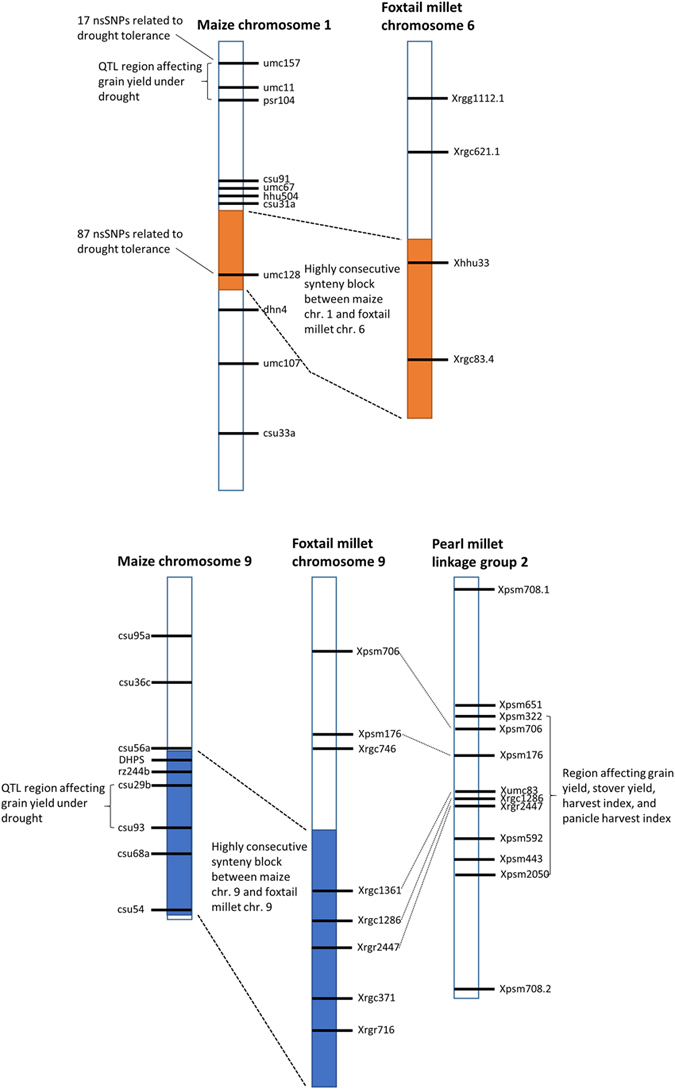
Summary of regions related to growth, grain yield and drought tolerance between maize, foxtail millet and pearl millet. Chromosomes and linkage groups between species do not share the same scale due to magnitude differences in genome size. Synteny blocks between millet and maize are represented in reverse order from SyntenyMiner to preserve reference genetic marker order and alignment representation.

Pearl millet (*Pennisetum glaucum*), another C_4_ millet, is slightly more studied as a model organism and, due to its popularity and more common consumption in India and parts of Africa, it is the most widely grown type of millet in the world. It also shares similar photosynthetic and growth traits with foxtail millet, such as the mechanism of C_4_ photosynthesis, variable grain yield and drought tolerance^31^. Currently, a complete assembly for the genome does not exist, but there have been several efforts on QTL and candidate gene discovery^33^,^34^. Since these efforts have been more developed on pearl millet than foxtail millet, and in view of the similarity between the two species, we collected the following mappings of regions that have been found to affect growth in pearl millet and how those regions map to foxtail millet, listed using their flanking genetic markers. More specifically, pearl millet linkage group 2 has a QTL region at the Xpsm322-Xpsm2050 marker position that has been found to be associated with increased grain yield, stover yield, harvest index, and panicle harvest index^35^. The region has also been found to have mappings to foxtail millet chromosomes 1, 4, and 9^5^. In foxtail millet chromosome 9 specifically, the pearl millet QTL region maps to a region covering the Xrgc1361-Xrgr 2447 marker position (Fig. 4). The Yadav lab has also made efforts in using gene-based markers to identify 13 candidate genes in these regions^36^. The candidate genes not only affect grain yield but also flowering time and leaf rolling (drought-related) traits.

#### Synteny comparison with *Zea mays*

Past studies in comparative genomics have shown that major QTL regions in *Zea mays* are syntenic orthologs with Setaria QTL^37^. We have made a synteny comparison using the transcripts of the TT8 assembly and that of the much better studied C_4_ species, *Zea mays*. Phylogenetic analysis suggests that the C_4_ grasses including maize, sorghum and sugarcane belong to one clade, while the C_4_ millet group including green millet (*S. viridis*), pearl millet and foxtail millet belongs to another clade^38^. Interestingly, all of these grass species employ NADP-malic enzyme as the major C_4_ acid decarboxylating mechanism to concentrate CO_2_ in the bundle sheath cells. The C_4_ carbon fixation pathway is a key pathway for drought tolerance that allows C_4_ grasses to maintain higher water/nutrient efficiency and to conserve soil moisture.

**Figure 5:**
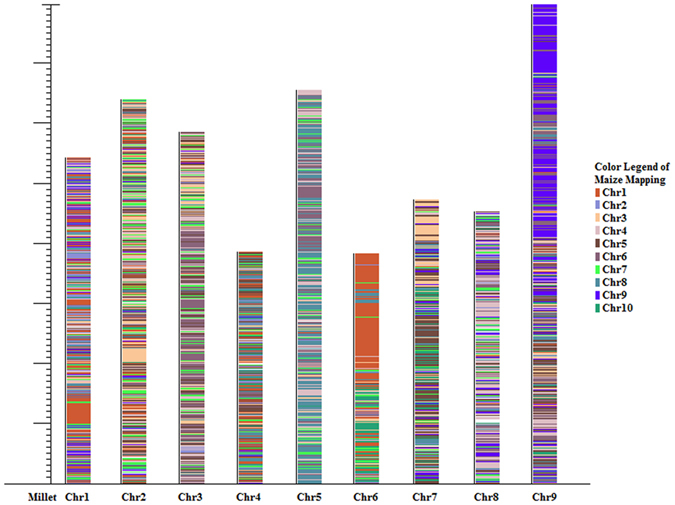
Synteny block comparison of *Zea mays* transcript sequences mapped to transcript sequences of the TT8 genome draft as a reference using SyntenyMiner (http://syntenyminer.sourcseforge.net/). Laid out on the graph are the nine chromosomes of millet with the colored regions representing the mapped maize chromosomes.

It has been estimated that the C_4_ millet clade split from the maize/sorghum/sugarcane clade ~27 Myr ago and that the maize genome is evolutionarily similar to the foxtail millet genome, but has undergone an additional round of whole genome duplication. In a previous paper, our team had categorized all known transcription factor genes and transcription co-regulator genes and made comparisons between maize and the millet annotation from JGI 8x v2 Sitalica_164 and Millet_scaffoldVersion2.3^39^. We used a *Zea mays* assembly from PlantGDB (B73_RefGen_v2) that contained 63,235 predicted genes from ten chromosomes with a total transcript length of over 96 Mbp compared to TT8′s 44,292 predicted genes from nine chromosomes with 49 Mbp total transcript length (Fig. 5). Of the 63,235 predicted maize genes, 271 candidate genes have been identified to affect drought tolerance^40^.

Although most millet chromosomes map to 3–4 maize chromosomes in non-continuous blocks, maize chromosomes 1 and 9 have continuous and consistent blocks mapping to millet chromosomes 6 and 9 consecutively (Fig. 5). Previous studies have shown that there are three QTLs on maize chromosomes 1, 5 and 9 that account for 75% of observed grain yield under drought phenotypic variability^40^. Maize chromosome 1 specifically has a QTL region towards the beginning of the chromosome at the umc157-psr104 position that has been linked to drought tolerance^41^. There are also 17 non-synonymous SNPs in that region that represent candidate loci related to the trait^40^. A second bin of 87 nsSNPs linked to the trait was also found towards the center of maize chromosome 1^40^. The TT8 synteny comparison shows that the synteny block is located towards the end of millet chromosome 6 and the center of maize chromosome 1, corresponding with the region of the 87 nsSNP bin of maize.

For maize and millet chromosomes 9, both synteny blocks are at the end of each chromosome. The synteny block on maize chromosome 9 corresponds to the QTL region affecting grain yield under drought in the csu29b-csu93 position^41^. This is in agreement with the growth factor proteins we annotated on millet chromosome 9 as well as the synteny with pearl millet linkage group 2 that shared the same traits (Fig. 4). Interestingly, comparative studies suggest that foxtail millet requires only 1/3 of the water required by maize for maintenance of normal growth^32^. How it achieves this high water efficiency awaits further analysis.

Since maize and foxtail millet are both NADP-ME subtype C_4_ plants we also conducted a synteny analysis specifically with the subtype-related genes. Of the 9 major NADP-ME subtype genes, we found the genomic organization of NADP-MDH genes matches the synteny mapping of maize chromosome 1 to foxtail millet chromosome 6. In general, however, the organization of the majority of these genes differs significantly (Table S7). The NADP-ME subtype genes were also found to have similar genome organization in JGI 8x v2 Sitalica_164.

#### Candidate gene models

Based on our syntenic analysis of QTLs and known regions affecting growth and drought in other species, and areas of novel assembly improvements in foxtail millet, we have identified eight gene models in the TT8 assembly related to growth factors, regulation or inhibition (Table 3). The majority of these putative proteins were hits from similar growth-related proteins in maize, and 50% of them are from chromosomes 8 and 9 of the TT8 assembly. These gene models are candidates for further studies and can be used to facilitate overexpression studies in future foxtail millet generations.

## Discussion

One of the common debates about using reference assembly vs. *de novo* assembly is the balance between too much bias from the reference genome and having a more complete genome^20^. Before we began assembly, we had mapped our raw PE reads to JGI 8x v2 Sitalica_164 and Millet_scaffoldVersion2.3 and noticed a 95% mappable rate, which means an assembly would most likely be very similar, and this has indeed been verified by our MUMmer plots. In view of this sensitivity and the need to preserve the novel information of the genome, we used *de novo* assembly to get the best assembly covering most of the genome before introducing reference information. This includes post-assembly improvements such as gap closing and scaffolding. In addition, 98% of the PE reads can be mapped to the final draft of the TT8 assembly, showing comprehensive presentation of the genome.

ALLMAPS improved the TT8 assembly from an N50 at 1,774,338 bp to 43,352,192 bp and was able to form nine large scaffolds that matched the nine chromosomes of foxtail millet. The development team of ALLMAPS had reported introducing translocation and inversion events to test the robustness of the tool and had shown that the tool was more susceptible to influence by inversion events^17^. Yet this did not impose a serious problem in the TT8 assembly, as most of the markers showed high alignment consistency for our ALLMAPS figure (Fig. 2).

The second major improvement was from reference-guided gap closing and extension. The strategy of making an insertion based purely on an alignment with JGI 8x v2 Sitalica_164 might lead to a separation of a gene as the insertion is based on where the gap takes place and not on biological annotation. It is also worth noting that none of the PE reads that were inserted to fill gaps did not originally assemble to scaffolds in those locations during ALLPATHS-LG assembly, nor did any scaffolds anchor to those locations during ALLMAPS assembly. However, there are still many unique GO terms and genes found in the regions which are not exclusively repeats.

In our annotation we identified several unique GO terms related to metabolic processes. We also annotated several growth-related genes on millet chromosomes 8 and 9. More so, the terminal region of chromosome 9 also mapped to pearl millet linkage group 2 at the Xpsm322-Xpsm2050 position and to maize chromosome 9 at the csu29b-csu93 position, genes in both regions known to correlate with grain yield. For drought tolerance, a large consecutive synteny block was identified between the chromosome 6 terminus of the TT8 millet assembly’s and maize chromosome 1 at the umc157-psr104 position. The latter region is known to contain a QTL for the trait, with several candidate genes identified, including AC232238.2_FG004, AC233953.1_FG005, and AC233961.1_FG001^40^. Drought tolerance genes for foxtail millet can be further identified and studied using expression level measurements and transcriptome analysis of the roots as done in a maize experiment on drought tolerant inbred AC7643^39^.

Although a desirable aim of crop studies is to pinpoint specific mutations and QTLs responsible for specific traits, the major accomplishment of our effort was to assemble a more complete genome and to provide insight into newly assembled regions, sequence variations and similarities to other closely-related species. For future perspectives, this comprehensive genome can facilitate further studies such as population genomics using MutMap^42^ and generational breeding^43^. It will also facilitate the identification of mutations responsible for novel trait improvement from the mutagenized pool of mutants. The more complete assembly and annotation work we present here serves as a foundation for those future discoveries.

## Materials and Methods

### Genomic DNA preparation

To prepare plastid-free chromosomal DNA of the TT8 cultivar (NCBI BioSample SAMN04534922), nuclei were first isolated from young leaves by gentle grinding in sucrose-based extraction buffer according to previous protocol (Construction of plant BAC libraries: An illustrated guide, http://www2.genome.arizona.edu/research/protocols). Genomic DNA was carefully released from nuclei in lysis buffer, treated with RNase A and proteinase K before purification through QIAGEN Anion-exchange Resin and Genomic DNA Buffer Set (Cat. 19060) under low salt and pH conditions. Genomic DNA was eluted in a high salt buffer and then concentrated and desalted by isopropanol precipitation. Genomic DNA was assayed for purity by NanoDrop (ThermoScientific, USA) and quantified by Qubit Fluorometer (Invitrogen, USA). The DNA integrity was checked by gel electrophoresis for major band at or larger than 40 kb.

### High throughput sequencing using multiple platforms

For a hierarchical *de novo* assembly of the foxtail millet genome, both Illumina and Roche 454 platforms were applied to construct NGS libraries containing various fragment sizes for primary assembly and scaffolding, respectively (Tables S1–S4).

To establish the foundation of assembly, short paired-end libraries were constructed, using the TruSeq DNA Library Prep Kit (Illumina, USA) with four gel size selections, containing insert size at average 182, 273, 381, and 466 bps, respectively. To generate end-overlapping paired-end reads, the two shorter ones were sequenced on HiSeq2000 High Throughput Mode at PE2*100 and two longer ones on MiSeq at PE2*300nt, respectively.

For genome scaffolding, jumping data containing increasing insert lengths were constructed, using Illumina and Roche 454. A series of mate-pair libraries were constructed, using Nextera Mate Pair Library Preparation Kit (Illumina) according to the manufacturer’s protocol. Totally five libraries at size increments at 2–4, 4–6, 6–8, 8–10, and 10–15 kb were generated and sequenced on HiSeq Rapid Mode at PE2*150. To help fill in the small gaps occurred during assembly, additional TruSeq DNA paired-end libraries containing longer inserts were constructed with gel-size selection at 1 kb and 1.7 kb range, respectively, and subjected to Rapid Mode sequencing on HiSeq2000 at PE2*150.

To further jump across longer range, Roche 454 platform was applied to enable large gap size sequencing by constructing a 20~30 kb jumping library according to the 454 Paired End Rapid Library Preparation Manual (gel size targeted at 20–40 kb) and sequencing on GS FLX+ (Roche 454, USA).

To help bridge over gaps at 1–15 kb, Long-Read sequencing was explored by using the TruSeq Synthetic Long-Read DNA Library Prep Kit (Illumina). In brief, genomic DNA was sheared by Covaris M220 (USA) to the majority at 10 kb and gel purified at 8–20 kb range. The long fragments were ligated with linker and amplified by long-range PCR, and subsequently diluted and aliquoted into a 384-well plate at 3,000–5,000 fragments per well. Each pool was then converted into shotgun libraries by transposon tagmentation, using the Nextera DNA prep reagent in the SLR kit. After amplification, the libraries were pooled together, quantified, and sequenced on HiSeq2500 at PE2*100 (Rapid mode). The sequencing data were simultaneously transferred to BaseSpace for cloud computing (https://basespace.illumina.com/home/index). The reads were barcode-sorted, processed and restored to longer fragments via *de novo* assembly per barcode by the SLR module on BaseSpace, resulting in a read length distribution between 1–19 kb and an average read length of 5.3 kb and modal at ~8 kb.

### Data processing

We used SoapDenovo EC^44^ for error correction using a 139x coverage set of 100 nt high-throughput PE reads to generate a consensus. We then used Trimmomatic^45^ on the 250 nt and 300 nt PE reads with leading and trailing ends removed based on quality. PE reads shorter than a minimum read length of 36 were discarded. FastQC^46^ and SGA^47^ were used iteratively to check quality, using a standard Phred quality score and FLASH^15^ to check overlap compatibility. For the mate pairs, we used NextClip^48^ to clip adapters, remove possible contamination and filter reads shorter than a minimum read length of 25. 454 reads were converted to a PE read format using a custom script to remove linkers and pair up the corresponding reads. The SLR reads were generated via BaseSpace module (Illumina) and used as given.

### Assembly

We ran the assembly tools ALLPATHS-LG, MaSuRCA^49^, and JR-Assembler^50^ on our PE reads with MP reads set as jump reads for scaffolding. We found ALLPATHS-LG to be the most favorable as far as number of scaffolds and total genome length are concerned. After that we ran several post-assembly improvements such as gap closing with GapCloser^44^, GapFiller^51^, and FGAP, and scaffolding with SSPACE^52^ and SSPACE-LR^53^. We then used ALLMAPS for reference-guided improvements and custom tools for reference-guided extensions and gap closing. Finally, we used BLAT on repeat masked remaining scaffolds, using RepeatMasker^54^ to map with the TT8 assembly and to remove redundant scaffolds. BWA, CEGMA and QUAST^55^ were used iteratively for QC evaluation.

### Annotation

#### Gene prediction

For gene prediction we used the MAKER2 pipeline to predict genes in the TT8 assembly. We used JGI’s general feature format (GFF) annotation and the expressed sequence tag (EST) data of 61,706 sequences from JGI 8x v2 Sitalica_164 for the gene prediction training and SNAP and GeneMark for the gene models.

#### Functional annotation

The putative protein sequences from gene model prediction were run against the NCBI’s NR database by BLASTP. Blast2GO and InterProScan^56^ were then used to do functional annotation and retrieved GO terms with web services to corresponding databases for more detailed information on term, hit and category.

#### Comparisons with other assemblies and species

The published genome datasets were downloaded from JGI (JGI 8x v2 Sitalica_164) and BGI (Millet_scaffoldVersion2.3). The sequence variation comparison with JGI 8x v2 Sitalica_164 was done with custom frame reading software. We downloaded the maize assembly and annotation from PlantGDB (B73_RefGen_v2) and used SyntenyMiner for synteny block comparison.

## Additional Information

**Accession codes:** A BioProject and BioSample have been created for TT8 at NCBI with accession number PRJNA314430 and SAMN04534922, respectively. The raw reads have been submitted to the Sequence Read Archive with the following accession numbers: SRR3430957, SRR3431014, SRR3431170, SRR3431257, SRR3431335, SRR3431366, SRR3431368, SRR3431369, SRR3431370, SRR3431741, SRR3431742, SRR3431746, SRR3431747, SRR3431749, SRR3431750, SRR3431751, SRR3431752, SRR3431753. The assembled genome has been submitted to the Whole Genome Shotgun Sequencing project under accession number LWRS00000000, with analysis on predicted gene models with accession number SRZ147826.

**How to cite this article**: Tsai, K. J. *et al*. Assembling the *Setaria italica* L. Beauv. genome into nine chromosomes and insights into regions affecting growth and drought tolerance. *Sci. Rep*. **6**, 35076; doi: 10.1038/srep35076 (2016).

## Supplementary Material

Supplementary Information

## Figures and Tables

**Table 1:** Comparison among the TT8 assembly, JGI 8x v2 Sitalica_164 and Millet_scaffoldVersion2.3.

Statistic	TT8	JGI 8x v2 Sitalica_164	Millet_scaffoldVersion2.3
# scaffolds	2,688	336	3,269
Largest scaffold (bp)	65,039,919	58,970,518	4,589,508
Total length (bp)	477,547,503	405,737,341	423,359,136
N50 (bp)	53,212,001	47,253,416	1,007,752
N75 (bp)	42,684,089	40,408,058	572,782
N95 (bp)	39,546,705	35,964,515	123,677
L50	5	4	136
L75	7	7	265
L95	9	9	548
GC%	46.2	46.14	45.88
# N’s (bases)	3,381,668	4,826,887	26,194,380
# of gaps (sites of Ns)	7,136	6,455	34,282
CEGMA completeness	95.16%	94.76%	95.97%

**Table 2:** Comparison of predicted annotation results in three foxtail millet genome assemblies.

Annotation Category	TT8	JGI 8x v2 Sitalica_164	Millet_scaffoldVersion2.3
Predicted gene models	44,292	40,599 (24,000–29,000)	38,801
Sequence length of transcripts (Mbp)	49	46	39
BLAST hits (nr) with 10^−5^ e-value cutoff	33,897	34,901	32,042
BLAST hits (nr) with 10^−10^ e-value cutoff	32,630	34,419	30,920
Avg. align. score among BLAST hits (nr) (10^−5^ e-value cutoff)	529.8	620.6	539.8
Avg. align. score among BLAST hits (nr) (10^−10^ e-value cutoff)	548.1	628.4	557.3
Pfam hits with 10^−4^ e-value cutoff	39,098	28,012	24,004
Avg. e-value among Pfam hits	1.51e-6	1.4e-6	1.49e-6
Unique GO terms	1,962	1,304	1,164
Cellular Component terms	259	150	148
Molecular Function terms	895	665	587
Biological Process terms	808	489	429

**Table 3:** Eight growth-related candidate protein hits from homology search of TT8 assembly and their origin details.

Identification	Name	Species	Chromosome number	Protein length (aa)
GI:226529630 NP_001152607.1	Growth-regulating factor	*Zea mays*	1	229
GI:162461339 NP_001106023.1	Putative growth-regulating factor 4	*Zea mays*	2	273
GI:212724052 NP_001132314.1	Growth regulator	*Zea mays*	4	617
GI:226500274 NP_001150080.1	Growth inhibition and differentiation-related protein 88	*Zea mays*	5	363
GI:226510439 NP_001152557.1	Auxin-independent growth promoter protein	*Zea mays*	8	653
GI:226497922 NP_001147347.1	Cell growth defect factor 2	*Zea mays*	9	114
GI:162461280 NP_001104909.1	Indeterminate growth1	*Zea mays*	9	436
GI:18057158 AAL58181.1	Hepatocyte growth factor-regulated tyrosine kinase substrate-like protein	*Oryza sativa Japonica Group*	9	378
