# Supplementary material for: Assembling the Setaria italica L. Beauv. genome into nine chromosomes and insights into regions affecting growth and drought tolerance
Source: Sci Rep. 2016 Oct 13;6:35076. doi: 10.1038/srep35076 (PMC5062080; doi:10.1038/srep35076)
Supplement: Supplementary Information [file srep35076-s1.pdf]

SUPPLEMENTAL INFORMATION – TITLE AND AUTHORS

Assembling the *Setaria italica* L. Beauv. genome into nine chromosomes and insights into regions affecting growth and drought tolerance

Kevin J. Tsai[1,2], Mei-Yeh Jade Lu[3], Kai-jung Yang[3], Mengyun Li[3], Yuchuan Teng[3], Shihmay Chen[3], Maurice S. B. Ku[4,5*], Wen-Hsiung Li[3,6*]

[1]Bioinformatics Program, Taiwan International Graduate Program, Institute of Information Science, Academia Sinica, Taipei, Taiwan 11574

[2]Institute of Biomedical Informatics, National Yang-Ming University, Taipei, Taiwan 11221

[3]Biodiversity Research Center, Academia Sinica, Taipei, Taiwan 11574

[4]Department of Bioagricultural Science, National Chiayi University, Chiayi, Taiwan 60004

[5]School of Biological Sciences, Washington State University, Pullman, WA 99164, USA

[6]Department of Ecology and Evolution, University of Chicago, Chicago, IL 60637 USA

Corresponding authors' e-mail addresses: whli@uchicago.edu, mku@mail.ncyu.edu.tw

Corresponding authors' telephone: 001 773-702-3104; 886 5 271 7776

Other author's e-mail: kevin.j.tsai@gmail.com, meiyehlu@gate.sinica.edu.tw

SUPPLEMENTAL INFORMATION - TABLES

Table S1. Statistics of shotgun sequencing by Illumina paired-end sequencing. Total genome coverage by PE data is 234x fold relative to the assembled length at 477 Mb.

Library	Insert Length (bp)	Sequencing read length	Raw read count	Post-process read count	Post-process avg. read length	Post-process avg. overlap (nt)	Post-process coverage (477 Mb)
HiSeq PE	182	PE2*100nt	324,114,378	314,687,442	99nt x2	n/a	65.31
HiSeq PE	273	PE2*100nt	366,653,272	356,183,690	99nt x2	n/a	73.92
MiSeq PE	381	PE2*250nt	80,384,626	75,357,684	220nt x2	110	34.76

Table S2. Statistics of long insert paired-end and mate-pair sequencing data. Total genome coverage of the jumping data is 141x fold.

Library	Library insert/jumping size (kb)	Raw read count	Post-process read count	Post-process avg. read length	Post-process avg. insert length (bp)	Post-process coverage (477 Mb)
Long PE	1kb	177,977,566	177,977,566	150ntx2	800	55.97
Long PE	1.7kb	36,048,028	36,048,028	150ntx2	1,482	11.34
MP	2~4kb	69,416,020	38,449,898	97ntx2	2,714	7.82
MP	2~4kb	68,894,070	41,230,072	97ntx2	2,716	8.38
MP	4~6kb	70,970,522	39,005,998	97ntx2	4,714	7.93
MP	4~6kb	69,379,950	37,248,230	97ntx2	4,716	7.57
MP	6~8kb	47,026,350	26,248,048	101ntx2	6,716	5.56
MP	6~8kb	46,421,132	25,909,298	101ntx2	6,718	5.49
MP	8~10kb	75,759,594	38,636,414	96ntx2	8,714	7.78
MP	8~10kb	73,995,804	36,947,744	96ntx2	8,718	7.44
MP	10~15kb	70,470,368	39,149,822	96ntx2	12,214	7.88
MP	10~15kb	70,146,552	38,013,790	96ntx2	12,214	7.65

Table S3. Roche 454 long distance paired-end (LDPE) (20 kb jump) data.

Library	Raw read count	Post-process read count	Post-process avg. read length	Post-process avg. insert length
454 LDPE	1,284,306	710,310	174x2	19,452
454 LDPE	1,383,054	664,973	172x2	19,470
454 LDPE	1,347,125	698,754	177x2	19,456

Table S4. Illumina synthetic long reads (SLR) data of the millet genome.

SLR read length category	SLR count	N50 length (bp)	Avg. SLR read length
Length: 500~1499 bp	32,039	979	898
Length: ⩾ 1500 bp	97,425	6,669	5,326

Table S5. Comparison of the TT8 assembly through post-assembly improvement stages.

Statistic	ALLPATHS-LG	ALLPATHS-LG + Gap closing + Scaffolding	ALLPATHS-LG + Gap closing + Scaffolding + ALLMAPS	ALLPATHS-LG + Gap closing + Scaffolding + ALLMAPS + Reference-guided Gap close and Extension
# scaffolds	4,771	4,194	3,558	3,558
Largest scaffold	10,093,542	10,280,153	60,135,426	65,039,919
Total length	415,423,866	415,347,831	415,411,431	479,782,239
N50	1,435,189	1,774,338	43,352,192	53,212,001
N75	655,501	727,394	36,701,690	42,684,089
L50	78	64	5	5
L75	191	158	7	7
GC%	46.05	46.12	46.12	46.19
#N’s	45,586,082	20,897,537	20,961,137	7,847,864

Table S6. Repeat content comparison using RepeatMasker of major categories in three genome assemblies of foxtail millet.

Repeat Category	TT8	JGI 8x v2 Sitalica_164	Millet_scaffoldVersion2.3
Total repeat content	26.44%	26.15%	22.66%
Interspersed repeats	25.27%	24.96%	21.54%
Retroelements (interspersed)	17.88%	18.04%	15.06%
DNA transposons (interspersed)	7.16%	6.69%	6.25%

Table S7. Comparison of the major NADP-ME C4 subtype gene organization between foxtail millet and maize. CA, carbonic anhydrase; PPC, PEP carboxylase; NADP-MDH, NADP-malate dehydrogenase; NADP-ME, NADP-malic enzyme; PPDK, pyruvate, phosphate dikinase.

Name	Arabidopsis locus name	Maize chromosome	Maize position	Foxtail millet chromosome	Foxtail millet position
**βCA2**	AT5G14740	3	215,547,876	5	34,296,235
**ßCA4**	AT1G70410	4	168,867,433	9	19,994,763
**PPC2**	AT2G42600	8	173,256,053	2	29,827,781
**NADP-MDH**	AT5G58330	1	203,209,824	6	40,555,405
**NADP-ME1**	AT2G19900	3	201,756,871	5	39,652,675
**NADP-ME2**	AT5G11670	6	139,464,390	5	39,625,441
**NADP-ME3**	AT5G25880	3	201,756,871	9	52,371,643
**NADP-ME4**	AT1G79750	8	174,612,565	9	6,097,520
**PPDK**	AT4G15530	8	106,535,509	3	27,683,640

SUPPLEMENTAL INFORMATION - FIGURES

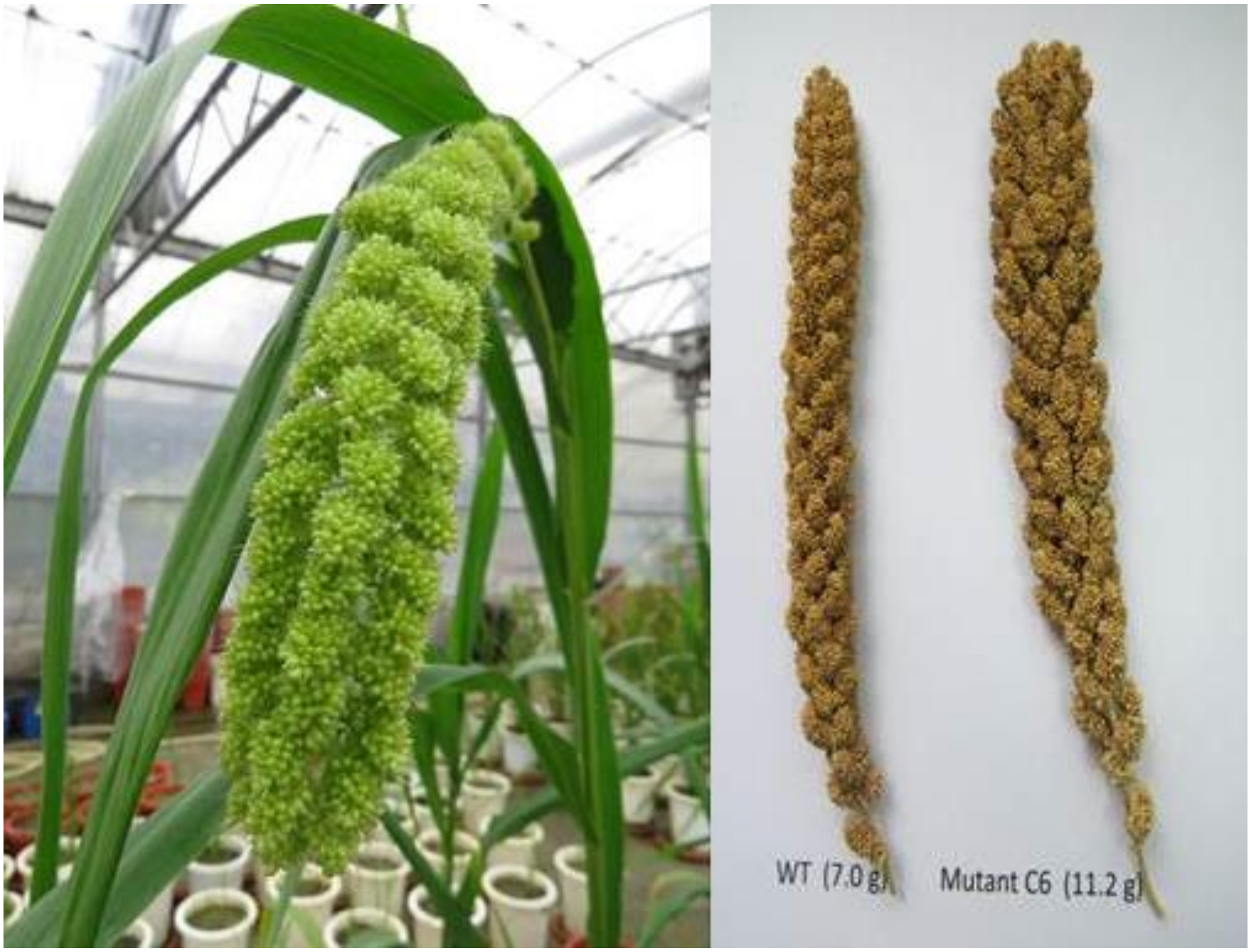

Figure S1. The panicle of the foxtail millet mutant (C6) isolated from the sodium-azide mutagenized mutant pool of the TT8 cultivar. The mutant produces a larger panicle with more and heavier seeds. The cultivar was grown in Taitung County, Taiwan.

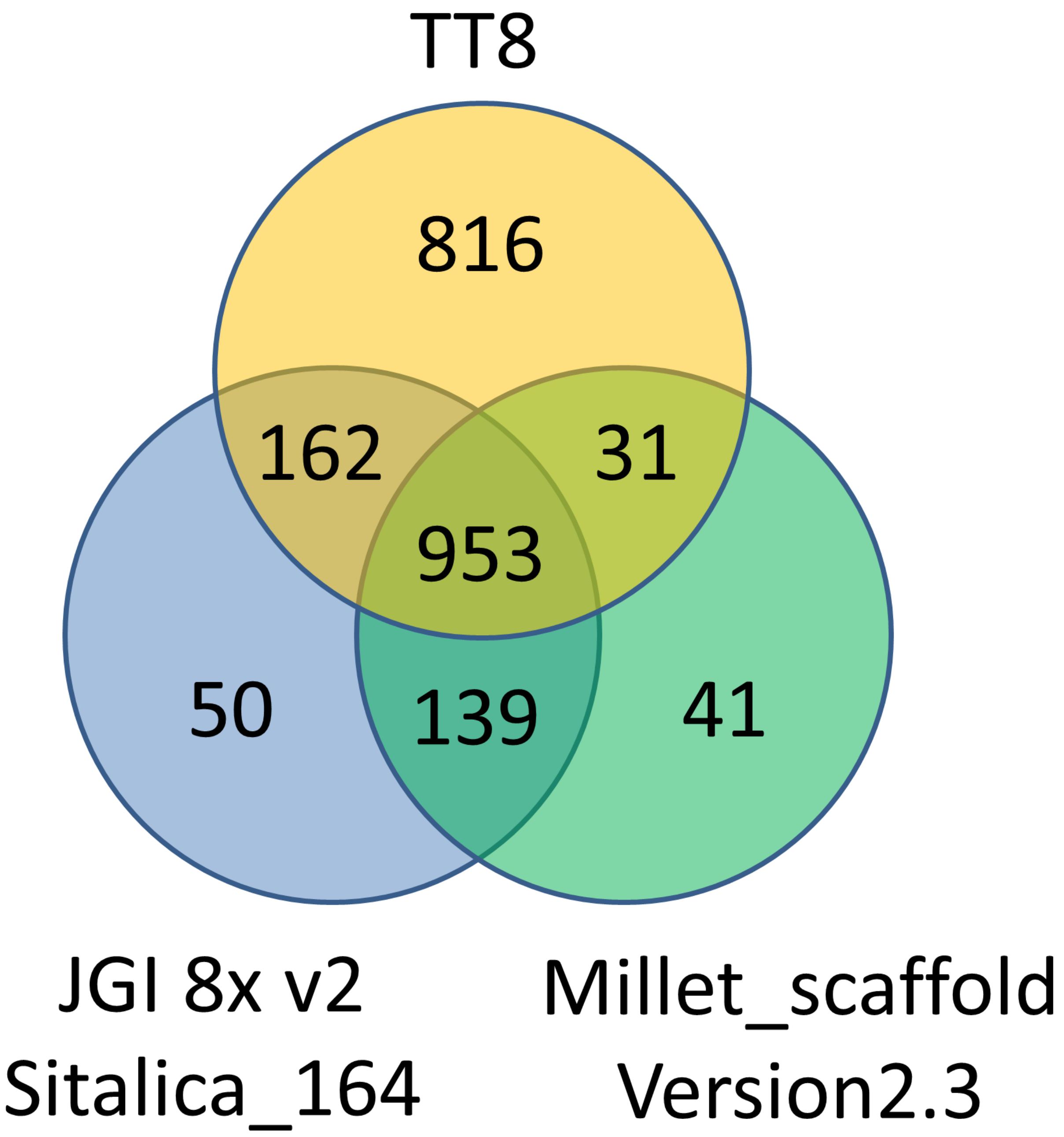

Figure S2. Venn diagrams to show the overlap of unique GO term exclusivity among the TT8 assembly, JGI 8x v2 Sitalica_164 and Millet_scaffoldVersion2.3.

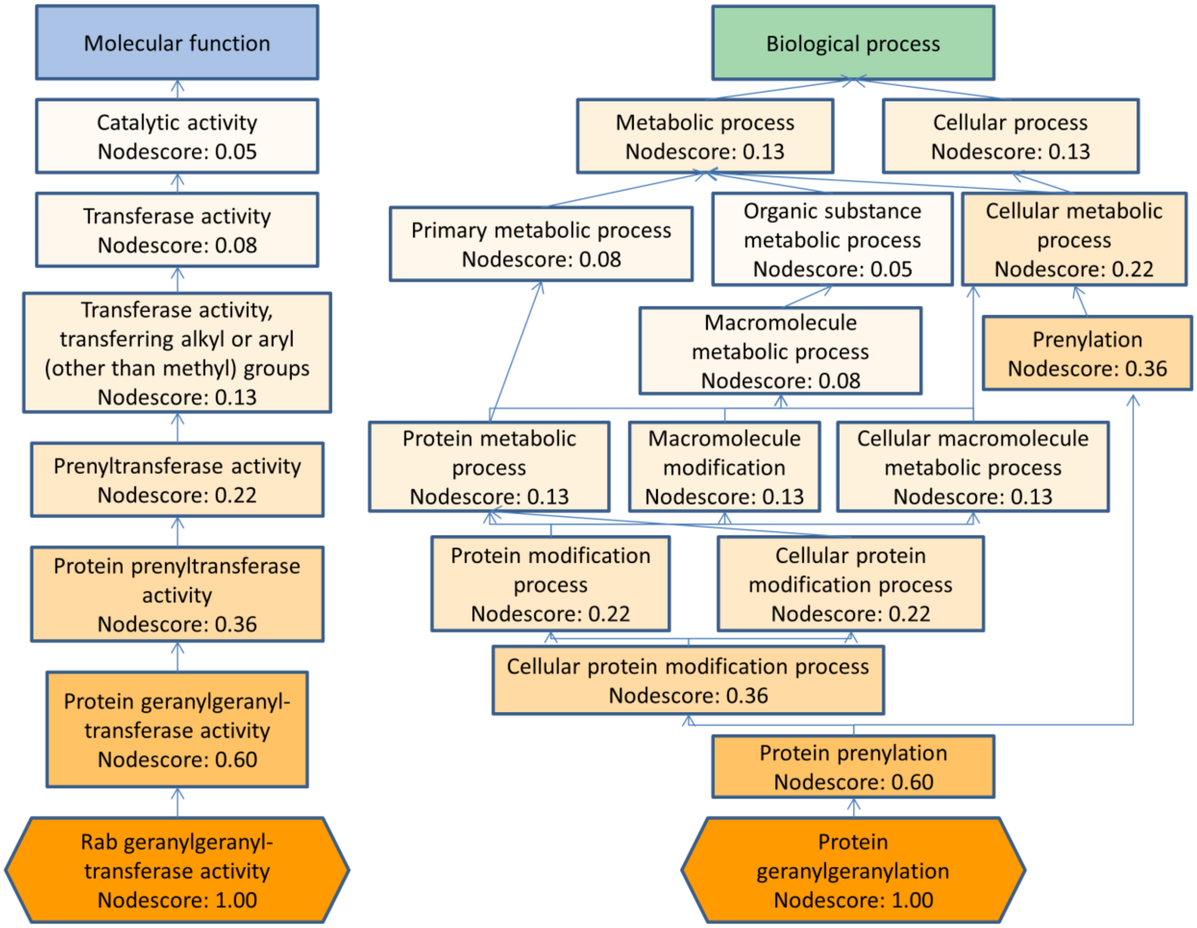

Figure S3. A functional annotation graph generated by data from Blast2GO (Conesa *et al.,* 2008) of 95 unique GO terms annotated from newly assembled regions. The node score takes into account the topology of the ontology and the number of sequences directly annotated to the node.

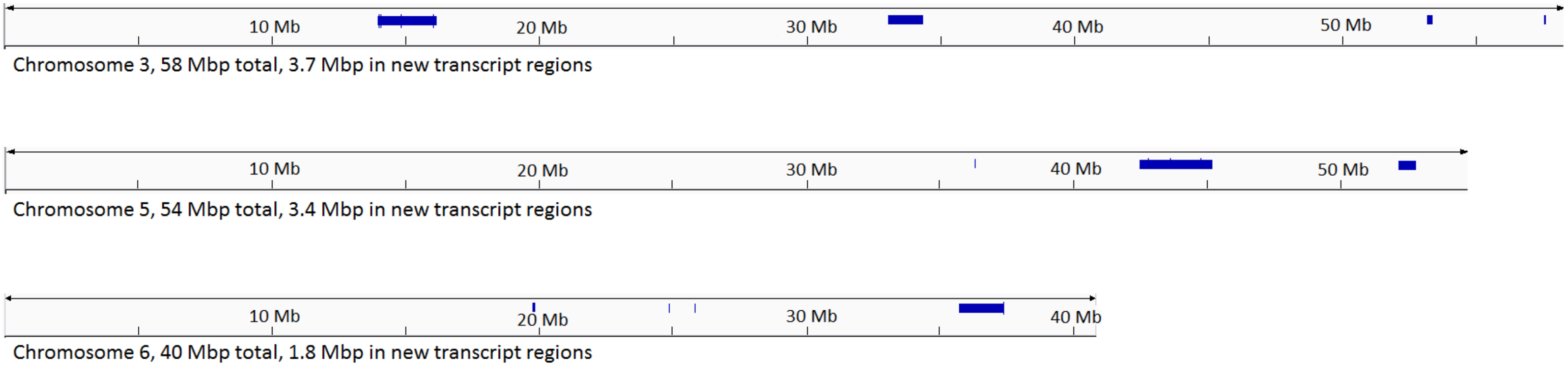

Figure S4. A chromosome view of chromosomes 3, 5 and 6 created by Integrated Genomics Viewer (Thorvaldsdóttir *et al.,* 2013) of the newly annotated genes found in regions of the TT8 assembly that were not assembled in previous assemblies. Transcript regions are indicated in blue.
